# Innovations in Social and Emotional Learning Research and Practice: Building from Evidence and Applying Behavioral Insights to the Design of a Social and Emotional Learning Intervention in Northeast Nigeria

**DOI:** 10.3390/ijerph18147397

**Published:** 2021-07-11

**Authors:** Rebecca Bailey, Natasha Raisch, Sonya Temko, Britt Titus, Jonah Bautista, Tahirat Omolara Eniola, Stephanie M. Jones

**Affiliations:** 1 Ecological Approaches to Social Emotional Learning (EASEL) Lab, Harvard Graduate School of Education, Cambridge, MA 02138, USA; nraisch@gse.harvard.edu (N.R.); sonya_temko@mail.harvard.edu (S.T.); stephanie_m_jones@gse.harvard.edu (S.M.J.); 2 International Rescue Committee, New York, NY 10168, USA; britt.titus@rescue.org (B.T.); jonah.bautista@rescue.org (J.B.); tahiratomolara.eniola@rescue.org (T.O.E.)

**Keywords:** social and emotional learning, Northeast Nigeria, education in emergencies, program design, behavioral insights, teacher voice

## Abstract

Recent studies suggest that social and emotional learning (SEL) programming has the potential to be effective in conflict-affected regions, yet evidence is limited, and findings to date are mixed. One hypothesis about why SEL interventions in education in emergencies (EiE) settings have not generated the anticipated results is that the SEL content and materials have not been sufficiently localized to the context, leading to poor cultural relevance or fit. A second hypothesis is that SEL program demands tend to be high and capacity for implementation is low, undermining sustainability and impact. The current study addresses these challenges by investing in locally driven SEL content and design as a way to ensure that SEL materials are grounded in local values and needs, culturally appropriate, relevant to the specific context, and feasible to implement. The study draws on the developmental and prevention sciences as well as the field of behavioral insights to test evidence-based interventions intended to encourage desired behaviors around uptake and implementation. This paper documents the activities conducted during the project’s design phase, including landscape research, creation of initial prototypes, design workshops and rapid prototyping, and field testing. Findings suggest that using local values, practices, and framing in SEL programming increases relevance and appropriateness for the Northeast Nigeria setting. Furthermore, targeted behavioral insights interventions increased the uptake, habitual and regular use, as well as correct implementation of SEL activities. The findings contribute to the emerging literature on the cultural variability of SEL and the need to consider the context when designing and implementing programs in any setting.

## 1. Introduction

Broadly speaking, social and emotional learning (SEL) refers to the process through which individuals learn and apply a set of social, emotional, cognitive, and related skills, as well as attitudes, behaviors, and values that help direct their thoughts, feelings, and actions in ways that enable them to succeed in school, work, and life [1,2]. Social and emotional skills are a foundation for positive development, contributing to key outcomes across learning, health, and wellbeing [3,4,5]. Research demonstrates that classrooms function better and students learn more when children have the skills to plan and organize, focus attention, manage emotions, persist in the face of difficulty, and navigate relationships with adults and peers [6,7,8]. High-quality social and emotional learning (SEL) interventions are shown to improve school liking, attendance, and mental health outcomes, in addition to academic achievement [9,10,11,12]. SEL may be especially important for children exposed to adversity, as social and emotional development is sensitive to the negative effects of stress and trauma [13,14,15,16,17]. In addition, research indicates that high-quality SEL interventions can improve children’s skills and buffer them from some of the negative effects of adversity [18,19], and some studies find that SEL programs have their largest impacts among children and youth who face the greatest number of risks [20,21].

Based on this body of evidence, SEL is increasingly pursued within global education in emergencies (EiE) settings as a strategy to improve both learning and wellbeing outcomes among the world’s most vulnerable children and youth [22,23,24,25]. In the past 5 years, numerous multi-lateral agencies have released policy guidance calling for the integration of SEL into EiE settings [26,27,28,29]. Despite a growing interest in SEL from the EiE community, the current evidence base for SEL programming has been generated primarily in white, educated, industrialized, rich, and democratic (WEIRD) societies, which do not reflect the social or physical realities of the vast majority of the world and therefore should not be assumed to be representative of other populations and communities [30]. As a result, little is known about whether and how the benefits of SEL programming will hold true across diverse international and cultural contexts and whether the specific SEL constructs, mechanisms, and approaches developed or studied within western and stable contexts can be replicated or applied successfully to EiE settings.

Recent studies suggest that SEL programming has the potential to be effective in conflict-affected regions, yet the evidence is limited, and findings to date are mixed. A randomized trial of a universal school-based SEL program in the Democratic Republic of Congo found positive impacts on student academic learning and on student perceptions of some aspects of school and classroom climate, but no overall impacts on student wellbeing or mental health [31,32,33]. A randomized trial of SEL programming among internally displaced and refugee children in Niger found positive impacts on student math and literacy scores and overall grades, but no impacts on social and emotional outcomes [34,35]. In a randomized trial among Syrian refugees in Lebanon, children with access to SEL programming showed improvements in reading and math as well as some social and emotional outcomes—including increased positive perceptions of school climate, improved behavioral regulation, anger dysregulation, and sadness dysregulation, and reductions in hostile attribution bias; but no impact on mental health outcomes such as depression or anxiety, and unexpectedly, students with access to SEL programming experienced an increase in school stress [36,37,38]. While these findings consistently point to SEL as a mechanism to improve student learning in EiE contexts, more research is needed to understand the potential benefits for children’s social and emotional, mental health, and wellbeing outcomes.

These mixed findings may partly result from challenges in the design and implementation of SEL programs across diverse EiE contexts. One particular challenge is that many SEL programs are not locally developed, and therefore, local community needs, values, strengths, and voices are not represented. Researchers have raised concerns that the skills taught or measured in SEL programs may target social behaviors and cultural norms that are not appropriate or relevant for the children and communities using them, and may require significant local adaptation [39,40,41]. For example, the timing and relevance of specific SEL skills can vary across cultures and contexts, as can the key conditions and processes for effective implementation [42,43,44]. Furthermore, different communities may have a different language, framing, and familiarity with SEL-related concepts. One hypothesis about why SEL interventions in EiE settings have not generated the anticipated results is that the SEL content and materials were not sufficiently localized to the context, leading to poor cultural relevance or fit. 

A second challenge is that traditional evidence-based SEL programs typically rely on comprehensive and scripted curriculum. These programs are time- and resource-intensive and are difficult to implement as designed even in stable and well-funded contexts [45]. Research indicates that implementation is an important moderator of program impacts [46,47,48]. Specifically, higher dosage, fidelity, participant responsiveness, and quality of SEL implementation are associated with better student outcomes [9,49,50]. Yet, implementation challenges are exacerbated in EiE settings, where teacher turnover is high, literacy rates are low, and the resources needed to train and support staff are extremely limited [51,52,53]. Thus, a second hypothesis about why existing SEL interventions in EiE settings have not generated the intended results is that program demands tend to be high and capacity for implementation is low, undermining sustainability and impact. Combined with a low dosage, short duration, and/or low teacher or student attendance reported in some previous EiE studies [34,38] students may not have had sufficient exposure to quality SEL programming to produce measurable changes in the intended mental health and wellbeing outcomes. While some implementation factors are likely to be a challenge in all crisis-affected contexts, SEL programs can be intentionally designed to support better implementation, especially that tied to buy-in, uptake, and feasibility within EiE settings.

The current study was designed to address these two challenges by investing in locally driven SEL content and design as a way to ensure that SEL materials are culturally appropriate, relevant to the specific context, and feasible to implement. Our goal was to address the shortcomings of previous research by (a) building on a proof of concept for SEL Kernels [54] as an evidence-based yet adaptable approach to SEL, and (b) applying behavioral insights to the development, contextualization, and field testing of SEL materials in local EiE settings. SEL Kernels are a low-cost, flexible, evidence-based approach to SEL that can be tailored to meet the needs of local teachers and classrooms [54]. Insights from behavioral science can be used to identify and address barriers and bottlenecks to the implementation of a program or intervention. We also drew upon tools from human-centered design, which involves the end-user in each step of the design process, to iteratively test and refine ideas or assumptions, for example, through rapid prototyping.

This paper describes the process used to bring these complementary approaches together, with the goal of creating evidence-based and localized SEL materials for Northeast Nigeria through rigorous design and field testing research. We build on the growing body of literature about SEL in EiE settings and address the following research questions: first, what are the local needs, values, beliefs, and existing practices that shape how educators and parents in Northeast Nigeria think about children’s social–emotional development, and what language and framing are used by teachers, parents, and other local stakeholders to describe SEL-related concepts? Second, what SEL strategies, routines, or activities are useful and feasible for teachers in Northeast Nigeria school contexts? Third, what design, format, and delivery options work best to increase uptake and implementation of activities among teachers in Northeast Nigeria? In the following pages, we describe our methods and findings, including the specific features of SEL content, design, framing, and delivery mechanisms that were tested throughout the 18-month design research phase of this project, and how our results inform the final intervention materials. This study provides evidence for the use of innovative and systematic methods to design and test for local relevance and feasibility of SEL materials. Rigorous design, field testing, and implementation research is a critical step toward filling the knowledge gap about what works for SEL in global EiE settings.

## 2. Materials and Methods

### 2.1. Study Background

The United Nations International Children’s Emergency Fund (UNICEF) [55] reports that over 10.5 million children across Nigeria are out of school. In the Northeast region of Nigeria, where the state’s conflict with Boko Haram is centered, over 1 million conflict-affected boys, girls, and adolescents with no access to basic quality education or vocational training skills qualify as Persons in Need [56]. The education system in the northeast faces many barriers to providing a quality education, which are illustrated in a recent needs assessment carried out by the Education in Emergencies Working Group in Borno, Yobe, and Adamawa states. The needs assessment indicates that school infrastructure is strained to absorb students: in Borno, the functioning classroom-to-student ratio is 1:129. Teaching and learning materials are scarce: in Yobe, only 29% of schools report having enough materials for students. More than a third of schools report that 50–100% of their workforce does not regularly attend, as teachers face many attendance barriers themselves, such as illness [57]. The International Rescue Committee (IRC) provides support in the region through an Education in Emergencies (EiE) program funded by the United Kingdom’s Foreign, Commonwealth and Development Office (FCDO). This program, implemented in Borno and Yobe states, served as an implementation setting for the current study.

#### 2.1.1. Study Context

In IRC’s EiE Nigeria program, out-of-school children attend an accelerated learning program (ALP) intended to mainstream them into formal schools, and low-performing in-school children attend catch-up tutoring sessions. In both interventions, IRC provides teachers with teaching and learning materials, face-to-face training on content and pedagogy, coaching, and peer-learning opportunities through teacher learning circles. IRC’s curriculum consists of literacy, numeracy, and social and emotional lessons delivered in 45 min blocks. Children attend the program for 3 h a day, 5 days a week for 9 months. Findings from an IRC evaluation found that the ALP program had statistically significant, positive effects on children’s numeracy and reading skills, but with regard to SEL, the program showed impacts for only one outcome: children’s tendency to use aggressive conflict resolution strategies. Impacts on six other SEL outcomes showed null effects. Follow-up interviews conducted with teachers, coaches, and children revealed that many considered SEL the hardest portion of the curriculum to implement and cited the newness of the subject as a challenge [58]. The social and emotional lessons were scripted and complex and used unfamiliar SEL frameworks and terms, such as language and definitions from the CASEL framework [59]. They also contained some activities that were difficult to implement in the context of Northeast Nigeria, for example, because they required materials that teachers did not have access to or asked children to move in ways that were uncomfortable given small classroom spaces or hot weather. For this reason, teachers reported skipping lessons, which resulted in low levels of implementation. Local education stakeholders were interested in developing new locally informed materials in order to support teachers’ understanding and use of SEL in hopes of ultimately improving student outcomes.

#### 2.1.2. Study Timeline and Participants 

The current study is a Stage 2 (Test and Position for Scale) project funded by the United States Agency for International Development’s (USAID) Development and Innovation Ventures (DIV). The project has three phases; the first phase is local input and co-design, the second phase is an implementation study, and the third phase is analysis and dissemination of findings. The original project timeline allocated 9 months for local input and co-design; however, due to COVID-related school closures, we extended the design phase. While school closures presented many challenges, the shift in plans enabled us to spend extensive time with a group of “core teachers” and necessitated that we try different methods and modalities, providing important insights for our design and field testing research. This paper describes the design phase, which ultimately lasted 18 months, and during which we conducted landscape research, created initial prototypes, facilitated design workshops, and engaged in field testing with local teachers in NE Nigeria. 

Landscape research took place between December 2019 and January 2020. There were 145 participants in semi-structured interviews, focus groups, and co-creation workshops, made up of teachers (66%), caregivers (29%), and government officials (5%) in Borno and Yobe states. Of these participants, a slight majority were female (57%) and from Borno state (67%). Between February 2020 and January 2021, 12 teachers, who were part of IRC’s EiE tutoring program, participated in design workshops and multiple rounds of iterative field testing. We refer to this group as the “core teachers” due to the extensive feedback they provided throughout the duration of field testing. The final round of field testing, which began in October 2020, included an additional 13 teachers who were new to SEL and unfamiliar with the study. An equal number of male and female teachers with an average of 7 years of teaching experience participated, from Yobe and Borno states. They spoke Hausa as their primary language (in addition to English and other local languages) and were recruited through purposeful sampling. Regarding the sample size, it is worth noting that the purpose of user testing is not to include a representative sample as in traditional qualitative research, but to examine program assumptions with the real end-users of a program before beginning implementation.

### 2.2. Study Approach

The objectives of the current study are to: (1) identify local needs, values, beliefs, and existing practices around SEL in Northeast Nigeria and use that knowledge, in conjunction with the evidence base and local language, to create and frame locally relevant SEL materials; (2) identify strategies, routines, and activities that are feasible to implement in the Northeast Nigeria education context and generate evidence of usage and user-demand; and (3) test design, format, and delivery models that can be applied to increase uptake and implementation of the activities among teachers in Northeast Nigeria. These objectives address the primary research questions listed above. In order to pursue these objectives, we brought together two complementary approaches: SEL Kernels of Practice and Behavioral Insights. This is the first time these two approaches have been used together to inform the design and testing of SEL programs.

#### 2.2.1. SEL Kernels of Practice

By way of definition, kernels are simple procedures that affect a particular change in behavior [60]. Kernels are based on the assumption that effective prevention programs include multiple components—some of which are responsible for changes in behavior and others that may not be necessary for improved outcomes. By identifying these fundamental drivers of behavioral change, it is possible to create simple, low-cost, effective, and scalable strategies that influence specific outcomes or behaviors. Kernels typically require little-to-no resources [60], and therefore may be more feasible to implement than traditional programs, particularly within EiE contexts. As the “essential active ingredients” of more comprehensive programs, they have been found to be effective across a range of learning, health, and behavior outcomes [60,61].

Over the past 7 years, the Ecological Approaches to Social Emotional Learning (EASEL) Lab at Harvard University has been developing and testing a kernels approach to SEL within school and community settings. In order to identify common elements across effective SEL programs, we developed a coding system based on a comprehensive review of relevant developmental and prevention sciences literature, identifying key social and emotional skills tied to positive outcomes for children and youth [62,63,64,65,66]. The codes include skills, beliefs, attitudes, and competencies such as the ability to identify emotions, understand social cues, resolve conflicts peacefully, focus attention, cope with difficulty, have a sense of purpose and self-worth, and build and maintain supportive relationships with others. We coded and analyzed over 40 evidence-based SEL programs used in early childhood, elementary, middle school, and high school settings. We coded these curricula for specific SEL skills and instructional strategies and built a database of activities organized by skill, age group, and instructional type. By identifying and pulling out common elements across evidence-based SEL programs, we developed “SEL Kernels of Practice”, a set of short and targeted activities that are intended to build specific SEL skills or classroom practices [54]. Agnostic to programs or curricula, SEL Kernels can be adapted to fit different contexts and needs. 

#### 2.2.2. Behavioral Insights

Behavioral science is based on theories and evidence from psychology, economics, neuroscience, and other social sciences and provides a systematic approach to understanding human behavior and decision-making. Even in stable contexts, people do not always take up, engage with, or follow through with programs, despite the best efforts of the program designer. Behavioral science has shown that humans often struggle to convert their intentions into actions in predictable ways [67]. Introducing new programs is even more challenging in emergency contexts, such as Northeast Nigeria, where teachers may be overwhelmed by myriad responsibilities, and both students and teachers have experienced more than a decade of conflict. 

To improve teacher implementation, we have drawn on insights from behavioral science by using evidence-based interventions to encourage desired behaviors around uptake of activities, habitual and regular use of activities, as well as the correct use of activities. First, to encourage teachers’ frequent and habitual use of activities, we used a behavioral insight called “goal setting.” Helping people make concrete, specific plans increases the likelihood that they will achieve their goal for using activities, especially if they write those plans down [68]. This intervention asked teachers to plan their practice in terms of when, where, and how many activities they planned to try each week. 

Second, to encourage uptake of all the activities in the set, we introduced a gamified checklist. The behavioral science evidence shows that seeing progress, receiving feedback, or getting symbolic rewards as a result of changing a behavior can further encourage the behavior [69]. Teachers write down each time they try a new activity and receive “badges” based on how many activities they try. We tested the hypothesis that helping teachers visualize their progress would encourage them to try new activities, and that providing symbolic rewards for doing so would promote repetition of the activities. 

Finally, to encourage fidelity of implementation and regular use of activities, we used SMS messages that split information about each activity into three bite-sized chunks. Evidence from behavioral science indicates that people learn more easily if the information is broken down into simple steps [70]. Additionally, the regularity of the texts provides reminders to center teachers’ attention on students and the activities [71]. We complemented these three interventions with other supports such as behaviorally informed teacher learning circles, certificates, and videos.

### 2.3. Study Activities, Methods, and Materials

#### 2.3.1. Landscape Research

The first step in the design process was Landscape Research, which included semi-structured interviews and focus group discussions with over 140 local teachers, caregivers, and other education and community stakeholders. The purpose of this phase of work was aligned with our first research question: what local needs, values, beliefs, and existing practices are related to supporting children’s social and emotional development, and what specific skills, language, and framing are used by local stakeholders to describe concepts related to SEL? We also sought to identify program outcomes that may be motivating to teachers. This phase of work was designed to address our hypothesis that the more familiar or contextualized the program, the more valuable it will be to teachers and community stakeholders, thus encouraging higher uptake. Focus groups and interviews were conducted by local Nigerian education program officers and measurement and evaluation officers, who led conversations in Hausa and other local languages using a semi-scripted protocol. Sample questions included “What does it mean for a child to grow up to be successful in life here?”, “What things do children need to learn to become good people?”, “What activities do you use to teach children these important lessons?”, and “What is the role of a teacher in your community?” These questions covered three themes: priority skills, local activities (e.g., games, songs) used to teach those skills, and teacher perceptions of SEL and of their own identities and aspirations. By gathering information about SEL-related needs, values, skills, and existing practices in Northeast Nigeria—and the language used to describe these ideas—we were better able to adapt the program activities to the local setting in the hopes of increasing the cultural fit of program materials. 

#### 2.3.2. Initial Prototypes

The second step in the design process was the creation of initial prototypes of SEL materials. The purpose of this phase was to adapt local activities and SEL Kernels to meet the local needs identified during the Landscape Research and respond to our second and third research questions: what SEL strategies, routines, or activities are useful and feasible for teachers in Northeast Nigeria school contexts? Additionally, what design, format, and delivery options increase uptake and implementation among teachers in Northeast Nigeria? The research team adjusted elements of local activities to strengthen SEL skill-building and adapted existing SEL Kernels to meet important contextual parameters. For example, in selecting and adapting activities for Northeast Nigeria, it was important to consider class size, varying literacy levels among teachers, very limited resources, and other challenges identified by teachers. Using those parameters, we chose the most promising SEL activities and adapted them to create initial prototypes. By designing initial prototypes to minimize implementation burden on teachers, we hoped to increase the likelihood that teachers would use the materials with high frequency.

#### 2.3.3. Design Workshops and Rapid Prototyping

The third step in the design process was local design workshops in combination with rapid prototyping and other human-centered design methods. The goal of the design workshops and rapid prototyping was to continue to explore our first and second research questions by eliciting feedback from teachers on an initial set of a) behavioral intervention drafts and b) SEL activity drafts in order to better understand how teachers selected, understood, and implemented the activities. This phase of work was designed to address our hypothesis that presenting information that is aligned to teachers’ preferences, capacity, and needs will lead to greater sustainability and, ultimately, impact. We shared initial prototypes with IRC education program staff, Ministry of Education officials, and, importantly, local teachers during in-person workshops in Northeast Nigeria. The prototypes were rough drafts that were designed to encourage critical and constructive feedback from the intended users and inform immediate changes. Teachers were asked to rank the prototyping activities (from most favorite to least favorite), to role-play the activities amongst themselves, and try the activities in their classrooms with students. This was an iterative process, and we revised the materials in real time (after workshops or observations), so we could give teachers new versions to try the next day in response to their feedback and preferences. We also performed quantitative analysis on data from teacher rankings over time to identify patterns in how activities were ranked after various rounds of revisions. The data collected during this step were intended to identify how well teachers felt the activities responded to the needs in their setting (through the ranking activities and ensuing conversations), and determine implementation feasibility (through the role-play exercises and classroom pilots).

#### 2.3.4. Field Testing

The final step in the design process was field testing, where teachers were asked to integrate the prototyped activities into their teaching routines and share feedback with the research team. The goal of field testing was to continue building upon insights gained from previous phases, gather feedback from teachers on a revised set of a) behavioral intervention drafts and b) SEL activity drafts, and collect data about teachers’ processes for selecting, understanding, and implementing activities in the classroom with students. Field testing activities were designed to further explore the second and third research questions: what SEL strategies, routines, or activities are useful and feasible for teachers in Northeast Nigeria school contexts? What design, format, and delivery options work best to increase uptake and implementation among teachers? Over a span of 9 months, 60 semi-structured phone interviews with teachers were administered, 143 teacher surveys were collected, 62 classroom observation protocols were completed, and 7 focus group discussions were conducted to gather feedback from teachers as they tested preliminary SEL classroom materials. This step took place in two rounds. The first round of field testing was conducted remotely while schools were closed due to COVID-19. During the first round, we distributed physical and digital sets of activities to the 12 core teachers who had participated in the prototyping and design workshops. Each week, teachers received a small set of activities to rank and try with the children in their homes, and we conducted phone interviews with teachers to gather feedback. 

Once COVID-19 restrictions lifted and in-person meetings became feasible, the second round of field testing employed new methods and engaged additional teachers. The second round of field testing included a series of workshops with the core group of teachers as well as 13 additional teachers who had no prior experience with social and emotional learning nor knowledge of the intervention and therefore provided new perspectives on the materials. In the workshops, teachers responded to and generated ideas for framing and naming the SEL Kernels, and we used word association exercises to uncover promising or problematic associations with certain terms. Teachers also shared feedback on visuals designed by a local illustrator. Finally, as in-person teaching resumed, we asked teachers to try the activities in their classrooms with students and conducted classroom observations. Prior to receiving the activities, teachers attended a pilot training session and were provided with a set of revised behavioral insights-informed supports (see Section 3.4), including (a) a goal-setting activity designed to increase uptake, (b) a checklist and “teacher badge system” designed to increase frequency of use, (c) a set of SMS “nudges” to promote implementation, (d) a teacher learning circle guide designed to improve fidelity, (e) a set of short demo videos in which local teachers demonstrate how they use the SEL activities in their classrooms, and (f) a certificate designed to promote uptake. The field testing phase concluded with focus group discussions that gathered feedback from both core teachers and new teachers about the materials and support they were given during field testing. Teacher interview and focus group data were analyzed qualitatively using emic thematic content analysis to highlight local values and perspectives. Data from classroom observations were analyzed qualitatively using etic thematic content analysis to assign each activity a ranking based on the degree of implementation fidelity observed. We considered these data sources together, drawing from our qualitative and quantitative analyses to score each activity on a variety of criteria. The scores were added to a matrix that was used to evaluate whether each activity should be adopted, adapted, or removed from testing. The phone interviews and focus group discussions were designed to gather data on the relevance and appropriateness of kernels for SEL skill-building in Northeast Nigeria. The surveys and classroom observations identified specific implementation challenges that teachers experienced in the classroom, how behavioral insights elements such as the checklist and SMS “nudges” influenced buy-in and uptake, and what additional support (e.g., training, teacher learning circles) could promote implementation capacity.

## 3. Results

The social and emotional content featured in the materials is derived from findings related to needs, key skills, priorities, values, cultural norms, terminology, and framing for the Northeast Nigeria context. The design, format, and delivery of the SEL materials is derived from findings related to the uptake of and engagement with the activities, plus a variety of behavioral insights-informed supports adapted for the Northeast Nigeria context. Across project activities, we centered teacher feedback and voices when selecting and adapting activity strategies and behavioral insights supports, and consider this a crucial lever for buy-in, uptake, engagement, and fidelity. 

### 3.1. Landscape Research

The behavioral science literature indicates that peoples’ choices are largely influenced by the way those choices are “framed” [72]. Specifically, the uptake of a program is influenced by the language that is used to talk about it, and framing can be especially impactful in changing behavior if we link the new, desired behavior to people’s existing identity, emotions, or values. We therefore paid careful attention to the skills that teachers and caregivers described, and the language they used to describe them. Additionally, contextualized SEL should focus on the skills that matter most to the populations that programs serve [41], so we included skills, names, and definitions generated by the local population. Throughout landscape research, teachers pointed to a variety of skills that they consider essential for children to succeed in life, in school, and in relationships with others. Most frequently, teachers in Northeast Nigeria indicated that activities should build students’ abilities to succeed in their studies. Teachers believe that these types of abilities help students reach their academic and life goals and link the associated behaviors with discipline or self-discipline. Teachers also indicated that activities should help students improve their relationships. Teachers linked many social behaviors with the importance of showing respect towards peers, teachers, parents, and elders. Teachers also hoped to foster students’ ability to reconcile conflicts with peers, which they saw as important for securing peace within and among communities. Based on the student outcomes and behaviors that teachers told us were important, and using locally generated language that teachers provided, we identified three primary target skill areas, or outcome domains, for SEL Kernels in Northeast Nigeria: self-discipline, respect, and tolerance, described in Table 1.

Each kernel’s activity is linked to one of these skill areas, and therefore, to a student outcome that teachers value. The activities were well-received by teachers, who perceived them as a response to demonstrated classroom needs, and appreciated the target skills as important for their communities. An important part of Landscape Research was the process of “mapping” skills identified as important in Northeast Nigeria onto technical definitions of skills found within the SEL literature. Unfortunately, the wide range of terminology used in SEL research and programs has led to concern and confusion in the field [73,74]. For example, researchers and practitioners often use the same term to refer to different developmental constructs or use different terms to describe the same construct [75]. Even the term “SEL” is used to mean different things, such as peace education, mindfulness, mental health, and psychosocial support, anti-bullying or violence prevention, twenty-first-century learning, life skills education, and more. This confusion and lack of precision in SEL-related terminology can make comparisons of effectiveness across programs difficult [76] and ultimately undermines attempts to align research and evidence, programs and strategies, and assessment and evaluation [2]. Our goal in this study was to gather data from local stakeholders to identify skill areas and corresponding terminology that are important and relevant in Northeast Nigeria. Based on words and definitions provided by local stakeholders, we mapped the contextualized skill terminology onto technical definitions from the SEL literature to identify evidence-based activities that promote the skills that stakeholders identified as important. The mapping exercise ensured that the SEL kernels are rooted in evidence-based practices, while using context-specific language and framing for teacher- and student-facing materials in order to promote buy-in and uptake in the local setting. It is also important to note that we selected skills and created activities that responded to stakeholder needs yet did not reinforce aspects of the context that may promote inequality or hinder student development. Interviews with teachers helped us understand why they prioritized certain skills and activities over others, and based on those data, we held conversations with program staff to determine which skills and activities would provide the most benefit to students. Additionally, implementation data gathered during classroom observations helped us identify activities that promoted the participation of one gender over another, and we adjusted those activities and other support materials to encourage equal gender inclusion. Stakeholders also shared the skills they believed to be already well-represented in local teaching and child-rearing practices, and we decided to focus on skills that were identified as gaps or needing more support.

During the landscape research, we also learned about teachers’ perceptions of their own identities. During interviews, teachers described themselves not only as educators, but as role models who impart morals and values to children to help them meet their goals and succeed in school and life. The idea of teachers as role models informs the language used in the teacher training session, in which we recognize that teachers shape whom their students become, and we remind teachers that the activities are specifically designed to help them teach children the behaviors they need to succeed. This program framing is considered an important lever to encourage program uptake.

### 3.2. Design Workshops and Rapid Prototyping

**Figure 1:**
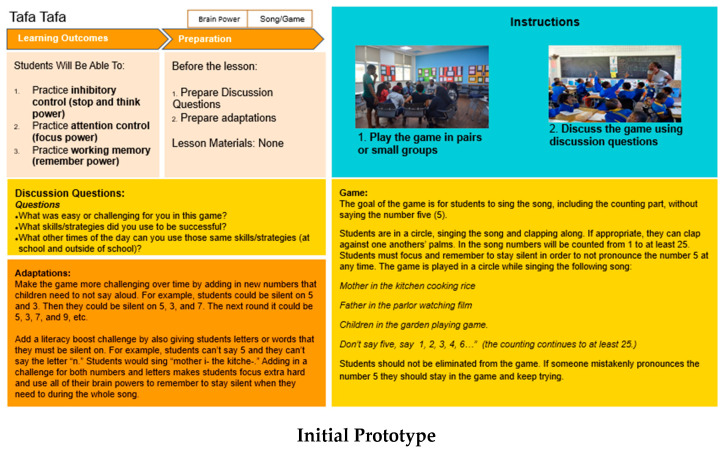

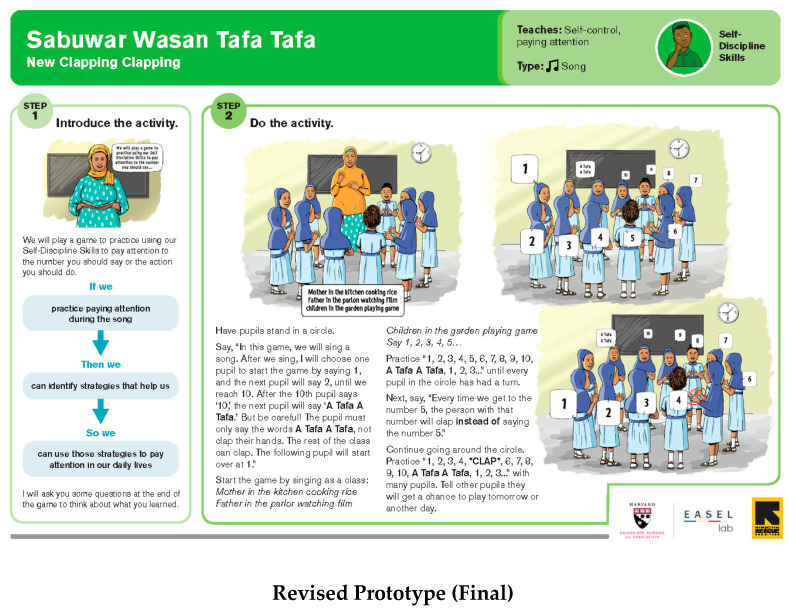
Initial and revised prototypes.

During the design workshops and rapid prototyping exercises, teachers were asked to rank the activities and tell us why and how they chose the activities they liked or understood the best. We also asked teachers to role-play the activities together without any background information, so we could understand what was immediately self-evident from the materials themselves, what assumptions teachers made, and what required additional clarification or support. Lastly, we asked teachers to try the materials in their classrooms with students, and local field staff conducted observations to determine what was engaging or challenging for students and teachers. The initial activity drafts included many complex components, various technical terms drawn from the literature on SEL, and stock photos to illustrate the activity steps (see Figure 1). During the design workshops, we learned that teachers found a large amount of text on the cards difficult to understand given varying levels of literacy and familiarity with English. Teacher rankings of the initial activities indicated that ease of comprehension and ease of use were the most important reasons for ranking an activity highly. Teachers valued activities that were simple and short, used familiar words, and were easy to understand, explain, and play. Additionally, teachers leaned heavily on the photos to support their comprehension of the activity, and images were often interpreted literally. This meant that photos needed to depict specific activity steps accurately; otherwise, teachers might implement the activity incorrectly. As we learned more about local needs, we revised the activity card prototype iteratively (see Figure 1). For example, we minimized the text and provided targeted guidance exclusively on areas that teachers requested (for example, a theory of change that explains exactly how students will practice the target skill and what behavioral outcomes teachers can expect). We simplified adaptation guidance into a bulleted list of tips and organized the card structure into three simple steps that are repeated across all activities. The photos on each card were replaced with locally designed illustrations that reflect Northeast Nigerian teachers and students performing specific aspects of the activity. Additionally, framing workshops allowed us to source words that reflect how teachers in Northeast Nigeria value and talk about SEL skills, and that terminology is included across the cards. 

### 3.3. Field Testing

During field testing, we collected feedback from teachers on multiple aspects of the activities, such as how challenging they were to understand or play, what skills children might learn from them, and what changes could make the activities better for students or teachers. These data indicated what components of the activities were working, what could be improved, and important levers for buy-in, uptake, and engagement. Through surveys and focus groups administered during field testing, teachers indicated that they valued activities that were locally sourced due to the high levels of engagement that they elicited from students. One teacher said, “I choose these [locally sourced activities] as my favorites because children really like them, already children are familiar with these activities and…with the new challenges that [come] with it, pupils always pay attention.” The familiarity of locally sourced activities seems especially important for uptake; in the last phase of field testing, the five kernels that were tried most frequently by teachers in their classrooms were locally sourced. One-third of the activities included in the final set is local strategies that were collected during the landscape research, and the remainder (drawn from a database of evidence-based SEL Kernels) include local elements such as stories and songs. 

We also learned that activity alignment with academics is important for uptake. Activities that were consistently ranked highly included elements of literacy and numeracy, and teachers mentioned that they would be more likely to use activities that helped them deliver their literacy and numeracy lessons. To respond to this feedback, we provide many ways to incorporate activities into academic lessons with tips to link the activity to literacy and numeracy learning objectives. 

**Figure 2:**
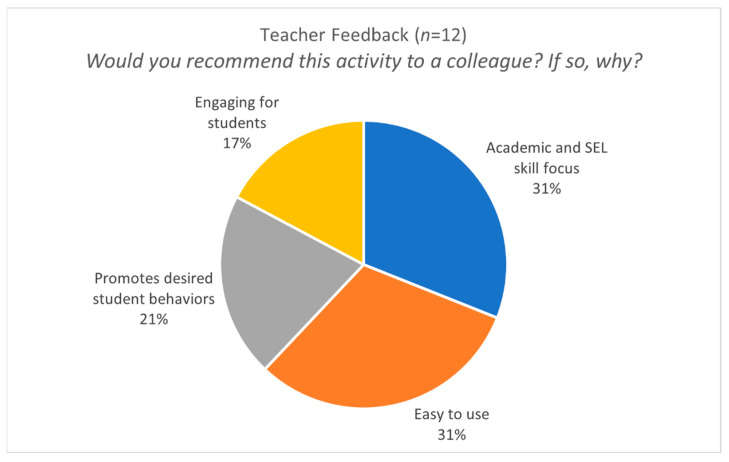
Summary of teacher reasons for recommending activities to colleagues.

Throughout field testing, teachers’ top-ranked activities also promoted student behaviors that were important to teachers, such as higher participation and collaboration. The survey that we used to collect feedback from teachers included a variety of questions about uptake. For example, one question asked teachers if they thought their colleagues would want to use this activity in their classrooms. When teachers responded affirmatively, the reason given most often was because the skills the activity targeted would promote desired student behaviors (see Figure 2). To that end, we included a support card to help teachers understand which activities they can use depending on the student behaviors they seek to promote in their classroom. For example, if a teacher wants to promote collaborative behavior, she could practice strategies that target prosocial behaviors and remind students to use those skills during group work. 

As we collected teacher feedback on activities throughout field testing, accounting for cultural norms around gender and considering how children of different genders would interact with the activities was particularly important to promote maximum, equal participation in the classroom. For example, teachers mentioned that some of the locally sourced activities were traditionally played by one gender more than another, which presented challenges when trying to encourage equal participation in the classroom. Teachers also indicated that, to satisfy gender norms, boys and girls should be able to play the games separately. Teachers adapted activities in various ways to satisfy gender norms, such as asking students to play at their desks or by row instead of making a circle or alternating playing the game with female and male participants. These adaptations were noted and shared with the participants in the teacher training session, and popular adaptations were explicitly mentioned in the activity card text. 

Field testing proved critical because some insights can only be gathered by observing how the end-users engage with the prototype (including its format, delivery, and content), which is often not possible through interviews or other qualitative methods. For example, teachers *told* us in interviews that they would like to receive SEL activities in SMS format. However, when we tested sending SEL activities in SMS format, we learned that teachers tried to print these messages onto paper or spent significant time writing them out by hand. When asked why, teachers explained that it is essential to have physical paper copies to conduct the activities. Field testing enabled us to learn that digital elements were useful support mechanisms, but should not replace the physical paper copies that teachers found so important. 

### 3.4. Behavioral Insights Supports

Throughout field testing, we used insights and tools from behavioral science to improve uptake and implementation fidelity. We designed a goal-setting exercise to encourage frequent activity use, and we found that asking teachers to participate in goal-setting prompts increased the number of activities they tried weekly. Teachers were asked to write a goal for the number of activities they want to try each week, and that goal was broken down into steps (for example, specifying the days and times they planned to try activities). When we tested sending a goal-setting prompt to teachers via SMS during remote field testing, we saw that the number of teachers using the activities that week increased nearly threefold. Building on that insight, we tested the support again when teaching resumed in-person, this time at the end of training in a paper goal-setting form. Before starting their goal-setting exercise, we told them that we had learned that the majority of the teachers we spoke to in the design phase had set a goal of trying an activity each day they had lessons with students. We know from the behavioral science literature that informing people of what others are doing, which reveals the hidden social norm, can be a powerful lever for behavior change [77]. Following this, almost all teachers planned to, and followed through with, using the activities every day. Teachers understand the purpose of the goal-setting exercise; one teacher said, “It is a part of planning, you set your goals and invest efforts to achieve your set goal.”

A gamified checklist was also piloted during field testing to encourage uptake and regular use of activities. This support links to a central idea of the kernels approach: the more frequently a teacher uses kernels in the classroom, the easier the activities will become for both teachers and students, and the greater the potential benefits for students. We also learned from testing that a potential behavioral barrier was uptake of only the most familiar activities instead of all the activities in the set. The gamified checklist shows teachers their progress, gives them symbolic awards for trying more activities, and appeals to teachers’ aspirational identities as role models for their students; the more activities they conduct, the more advanced they become. One teacher said, “Initially, both you and the learners are at [beginner] level because you are just starting the [activity]…as you progress you move to…the advance[d] stage. We can even explain this [activity] to our colleagues.” During field testing, teachers were also enthusiastic about the notion of tracking progress and improving. One teacher said, “The checklist helps us to see the progress we are making. We are all at the advance[d] level now.” Teacher engagement with the checklist was extensive: almost all teachers filled the checklist out every time they used an activity, and they tried an activity every day that they had a lesson. Teacher engagement with the checklist during field testing suggests it is a valuable tool for encouraging the uptake and recurring use of the activities. 

SMS as a delivery method was piloted during field testing to respond to the challenge of connecting with teachers remotely during COVID-19 school closures. Ultimately, sending SEL activities via SMS was shown to encourage uptake, and in some cases, fidelity and comprehension of activities. Initially, teachers told us that, due to unreliable energy and data sources, their phones were an unreliable channel through which to receive activities, and overall, they prioritized the paper format. However, teachers also reported that SMS provided a good reminder to perform the activities, and in our testing, we found that the hyper-simple SMS content sometimes facilitated teachers’ ability to identify and explain the skill focus of the activity. To continue testing the potential of SMS, a three-step SMS approach was designed to encourage fidelity of use, comprehension, and uptake. The justification for this approach is based on a study in which researchers found that sending SMS messages to parents three times a week (as opposed to once or five times a week) resulted in higher activity comprehension and uptake during a children’s literacy program [71]. By breaking information about each activity into three concise messages, we hoped to increase teacher understanding of the activity and skill and provide reminders for teachers to use the activities regularly. The first message provides teachers with a fact focused on the skill and links it to valued outcomes. The second message provides a tip focused on the activity that teaches that skill and provides details about how to use it. The third message encourages growth with ideas for adaptation (for example, by providing other ways to use the activity to promote skills or ways to make the activity easier or more difficult). The SMS nudge is intended to support—not replace—teachers’ use of the information on their physical copies of the activities. Teachers mentioned that receiving the SMS encouraged them to perform the activity more than once, each time building on the game by using the tips received. This support, when timed correctly, may be a valuable way to encourage teachers to try challenging activities they have not previously tried, in addition to providing teachers with extra guidance and encouraging growth for activities that teachers have already practiced.

The behavioral insights intervention portfolio also includes a series of teacher learning circles, a set of videos, and a certificate of program completion, which all reinforced the tested behavioral insights mentioned above. The teacher learning circles (TLCs) were designed as an opportunity for positive reinforcement from peers, which can narrow the gap between intentions and actual behavior by increasing program adherence and accountability. In the TLCs, we asked teachers to decide as a group which kernel activity was the most challenging for them and role-play it together. Teachers gave each other feedback and discussed tips for improving; overall, TLCs were observed to improve uptake, fidelity, and comprehension of the SEL activities. The videos were designed to support uptake and fidelity by providing demonstrations of a select number of more challenging activities. Teachers have said that watching the video improved comprehension and their willingness to try the activity. The certificate was designed to increase uptake of the activities by providing teachers with recognition from an entity that they value. We surveyed teachers during field testing to gauge their preference for certificate signee (for example, Nigeria Ministry of Education, International Rescue Committee, Harvard University, etc.), and teachers’ preference for a certificate signed by the Nigeria Ministry of Education indicates that potential collaboration with ministries of education, or other entities that are respected by teachers, could increase uptake of project materials.

### 3.5. SEL Product and Next Steps 

The design phase activities resulted in a set of activity cards, and a series of physical and digital behavioral insights informed support materials. The Kernels activity cards include 20 activities across three skill categories: self-discipline, respect, and tolerance. Of those activities, seven are locally sourced, and each activity includes local content and framing. The activities are provided in both English and Hausa. In addition, we created a portfolio of behavioral-insights-informed support materials, including a checklist, goal-setting form, and teacher learning circle guides, among other items. During teacher training, teachers receive a set of videos and later receive bi-weekly SMS messages throughout the duration of the implementation phase. Finally, teachers receive a certificate in recognition of their participation and progress. Next steps include an implementation study designed to generate broad evidence of user demand in Northeast Nigeria; we will study dosage, fidelity and quality of implementation, and participant responsiveness to the intervention. After the implementation study, we intend to revise materials based on findings and prepare for an impact evaluation to measure student- and classroom-level outcomes. If results from the impact evaluation are positive, we hope to bring the initiative to scale across the Northeast region of Nigeria.

## 4. Discussion

By applying innovative approaches to design research, we have identified a process for developing SEL Kernels that are deeply contextualized and respond to local values and needs as well as local implementation barriers. This process offers a more rigorous and systematic approach to the development and adaptation of SEL than traditional approaches, which tend to only revise, and at a bare minimum translate, surface elements when applying evidence-based programs in new contexts. Our hope is that this work will push the field forward to consider greater investment and more rigorous processes for design and implementation research, ultimately setting up programs to deliver on their intended outcomes and improve the quality of education overall in emergency and conflict-affected settings. Below, we discuss the limitations of our study and outline a set of recommendations for the field.

### 4.1. Study limitations 

The study’s limitations, especially regarding sample size and data collection methods, could impact the interpretation of research findings. First, our sample size was limited; although the teachers who participated in the design phase were recruited through purposeful sampling, only 24 teachers were involved due to resource and logistical constraints inherent in the project plan. Given that the goal of the study was to design a set of activities, the sample was not intended to be representative and the outputs are not intended to build generalizable knowledge. However, the involvement of more teachers during the design phase could likely have made our findings more generalizable to the intended population (teachers in Northeast Nigeria). Second, the field testing phase began just as schools in Northeast Nigeria closed due to the COVID-19 pandemic, which affected our data collection methods. Importantly, while schools were closed, teachers tested activities in their homes with their children instead of in their classrooms with students. Consequently, a significant amount of data gathered during field testing were collected remotely, and we were not able to conduct as many classroom observations as we originally planned. As teachers were testing activities in settings that differed greatly from their normal classroom environment, this may have affected their perceptions of the activities. Similarly, due to the nature of this remote testing, we were unable to ensure that all children involved in field testing were of the same age as the target population for the intervention, which could suggest discrepancies in the developmental fit of activities. Additionally, we necessarily collected teacher self-reports of activity implementation instead of observations conducted by trained measurement and evaluation officers. We caution against applying these findings too broadly for a number of reasons. This study’s intention is not to create generalizable knowledge but to define a process to create deeply contextualized SEL content that is responsive to local needs, values, and cultural norms and practices, as well as local implementation challenges and realities. Our findings derive from data collected through landscape research, design, and field testing activities that may be specific to the Northeast Nigeria context. Importantly, we do not yet know the ultimate impacts of our work, and implementation and impact research is needed in order to understand if the intervention leads to the high-quality implementation of SEL across the broader population and to measurable gains in child, teacher, or classroom-level outcomes. An implementation study is currently underway, and we plan to conduct an impact evaluation in the future to study SEL programming in this context.

### 4.2. Recommendations for the Field

Pairing an evidence-based set of SEL materials with local input through a systematic process of applying behavioral insights with user-centered design methods to the content, design, and delivery of the intervention resulted in a richly contextualized product and initial findings about SEL in the Northeast Nigeria context. While many elements of the final intervention are tailored to teacher and student needs in Northeast Nigeria, many of the core elements of the kernels approach remain consistent—for example, the focus on a target skill, the opportunity to practice and reflect on the skill, and the ability to adapt the activity to be used multiple times in different ways. The sustainability of the kernels approach is consistent with our theory and hypothesis, and melding the kernels approach with a systematic contextualization process is a way to bridge the field’s robust evidence on SEL with adaptations to make the work relevant for different places. As SEL is so deeply influenced by cultural norms, values, and practices, we should not expect identical results when using materials designed for one cultural or educational context in a different setting. However, we *can* pull from existing evidence (for example, the kernels approach) and push it in new directions via the processes described in this paper (for example, using behavioral insights approach and prototyping). While localization of SEL is critical, our findings suggest that it is also important to test specific assumptions about content, language and framing, format, and delivery in order to overcome barriers and to identify and harness drivers for implementation. Based on our findings, we provide the following points as considerations for thoughtful contextualization of SEL in diverse settings.

#### 4.2.1. Recognize Cultural Variability of SEL Needs, Values, and Framing 

While the need for SEL-related supports may be universal, the specific priorities and values tied to SEL programming are variable across contexts. Stakeholders who are removed from a particular setting may make assumptions about the values and skills that are important for that setting before (or sometimes, without) gathering input from the local population about their needs, skills, beliefs, and practices. This approach undermines the knowledge, capabilities, and strengths of local communities. In this project, centering local needs, values, and language enabled us to adapt evidence-based activities for cultural relevance. Implementation barriers and drivers of behavior are context-dependent, as well, so testing and contextualizing the behavioral interventions was critical for creating materials that were feasible and engaging for teachers. Using participatory research methods to select, describe, and frame SEL programming to reflect the local culture and context could be an important lever for increasing buy-in, uptake, and comprehension. Future research directions for this project include continuing to use participatory research methods to build an understanding of how skills are conceptualized, valued, defined, and manifested in Northeast Nigeria.

#### 4.2.2. Invest in Local Collaboration and Co-Creation 

Co-creation with local communities is a key pathway to identifying local needs and barriers to implementation, and ultimately, promoting program buy-in. First, teacher involvement as co-designers was essential to improving the appropriateness, usability, and uptake of activities. Teacher feedback became richer over time as trust was established and as we implemented their feedback in a continuous cycle, which made their contributions to the project tangible. We also provided teachers with stipends to recognize their efforts. Second, the collaboration and support of the IRC Nigeria country program were essential, as dedicated full-time staff served as the bridge whose knowledge, understanding, and familiarity with the context allowed for quality data collection, analysis, and interpretation. Importantly, the program staff understood how to pose questions to collect insightful, actionable answers, and subsequently, how to interpret the answers received and recognize factors that could influence responses. Although co-creation with local communities may require more time and resources than other design methods, these processes render highly contextualized insights that may favorably impact project outcomes.

#### 4.2.3. Prioritize Design and Implementation Research Before Testing Impact

Rigorous research should include design research, implementation research, and impact evaluations. To ensure relevance or feasibility in the context in which interventions are applied, the field of education in emergencies needs to invest in comprehensive design and implementation research *before* spending limited resources to test for impact. Design and implementation are essential phases that benefit from the same level of systemization that is applied to impact evaluation. The behavioral insights and human-centered design approaches provide a set of rigorous and systematic methods to lead this type of research and further the overall progress of the field.

## 5. Conclusions

This study was motivated by two hypotheses about why previous SEL programs in EiE settings have not generated expected gains in child-level SEL outcomes. The first hypothesis is that SEL content, design, and format of delivery may not have been sufficiently localized to the specific context. The second hypothesis is that teacher buy-in, uptake, and fidelity of implementation may have been low. In the current study, we used innovative methods for design and contextualization research of SEL materials in Northeast Nigeria, with an emphasis on local input to inform numerous aspects of the intervention. Specifically, we used a low-cost, flexible, and evidence-based approach to SEL and applied behavioral insights to the content, design, format, framing, and delivery of the intervention. Our paper describes a rigorous and systematic approach, which can be applied to SEL programs being developed, implemented, and studied in EiE contexts where existing evidence is limited and where local input and testing is required.

## Figures and Tables

**Table 1:** Primary skill areas.

Primary Skill Area (Local Terminology)	Importance (Local Terminology)	Associated Behaviors (Local Terminology)	Sub-Skills (EASEL Terminology)
Self-Discipline	Success in studies leads to achievement of academic/life goals	Paying attention to teachers, completing assignments autonomously, remembering lessons, following directions, and participating in class	Attention control, working memory and planning skills, and inhibitory control
Respect	Improved relationships lead to greater respect for others	Demonstrating friendly attitudes towards others; communicating, playing, and sharing with peers; demonstrating calmness and politeness	Prosocial and cooperative behavior skills
Tolerance	Reconciliation leads to peace	Less fighting in classrooms, demonstrating patience and tolerance	Conflict resolution and social problem-solving skills
